# MS2CNN: predicting MS/MS spectrum based on protein sequence using deep convolutional neural networks

**DOI:** 10.1186/s12864-019-6297-6

**Published:** 2019-12-24

**Authors:** Yang-Ming Lin, Ching-Tai Chen, Jia-Ming Chang

**Affiliations:** 1 Department of Computer Science, National Chengchi University, 11605 Taipei City, Taiwan; 2 Institute of Information Science, Academia Sinica, 115 Taipei City, Taiwan

**Keywords:** Peptide, Mass spectrum, Tandem mass spectrometry, Spectral library search, Protein identification, Machine learning, Deep learning, Deep convolutional neural networks

## Abstract

**Background:**

Tandem mass spectrometry allows biologists to identify and quantify protein samples in the form of digested peptide sequences. When performing peptide identification, spectral library search is more sensitive than traditional database search but is limited to peptides that have been previously identified. An accurate tandem mass spectrum prediction tool is thus crucial in expanding the peptide space and increasing the coverage of spectral library search.

**Results:**

We propose MS^2^CNN, a non-linear regression model based on deep convolutional neural networks, a deep learning algorithm. The features for our model are amino acid composition, predicted secondary structure, and physical-chemical features such as isoelectric point, aromaticity, helicity, hydrophobicity, and basicity. MS^2^CNN was trained with five-fold cross validation on a three-way data split on the large-scale human HCD MS^2^ dataset of Orbitrap LC-MS/MS downloaded from the National Institute of Standards and Technology. It was then evaluated on a publicly available independent test dataset of human HeLa cell lysate from LC-MS experiments. On average, our model shows better cosine similarity and Pearson correlation coefficient (0.690 and 0.632) than MS^2^PIP (0.647 and 0.601) and is comparable with pDeep (0.692 and 0.642). Notably, for the more complex MS^2^ spectra of 3+ peptides, MS^2^PIP is significantly better than both MS^2^PIP and pDeep.

**Conclusions:**

We showed that MS^2^CNN outperforms MS^2^PIP for 2+ and 3+ peptides and pDeep for 3+ peptides. This implies that MS^2^CNN, the proposed convolutional neural network model, generates highly accurate MS^2^ spectra for LC-MS/MS experiments using Orbitrap machines, which can be of great help in protein and peptide identifications. The results suggest that incorporating more data for deep learning model may improve performance.

## Background

Tandem mass spectrometry (MS^2^) has emerged as an indispensable technology in high-throughput proteomics experiments [1]. Tandem mass spectra generated from bottom-up proteomics consist of mass-to-charge ratios and relative abundances of a set of fragment ions generated from digested peptides. The patterns of these fragment ions are useful for the identification and quantification of proteomes in the sample.

There are two common approaches for protein identification: database search and spectral library search. The former searches each tandem mass spectrum (or MS^2^ spectrum) acquired from experiments against theoretical spectrums generated from all possible digested peptides (with trypsin in most of the cases) in the human proteome using a scoring function. The latter searches a MS^2^ spectrum against a spectral library, a collection of high-quality spectra of all identified peptides from previous experiments [2]. Although database search is more comprehensive and covers all possible peptide space, the sensitivity is lower because of the absence of intensity for each fragment ion in theoretical spectra. In contrast, spectral library search provides considerably higher sensitivity since a spectral library consists of realistic fragment ion intensities [3]. However, spectral library search is limited to peptides that have been previously identified, which hinders the application of spectral library search in areas where the discovery of novel peptides is of importance, such as the identification of peptides with mutations or peptides from isoforms of proteins. To take this into account, it is necessary to develop methods for computational prediction or simulation of MS^2^ spectra from amino acid sequences to expand the size of a spectral library.

There are several different strategies in predicting the MS^2^ spectrum of a peptide. MassAnalyzer, a pioneer work in computational prediction of a MS^2^ spectrum, uses a kinetic model on the basis of the mobile proton hypothesis to simulate peptide fragmentation [4, 5]. A semi-empirical approach is to predict the MS^2^ spectrum of a peptide from the spectra of similar peptides by peak perturbation [6]. The approach is based on the observation that the peptides of similar sequences produce similar fragmentation patterns in most cases. The concept is then generalized to a weighted *K*-nearest neighbor (KNN) approach in which a machine learning model first selects peptides that are likely to have high spectra similarity to the target peptide, and then a consensus algorithm combines their spectra to predict the MS^2^ spectrum of the target peptide [7]. Though the two approaches can yield good prediction accuracy for target peptides with similar amino acid sequence neighbors, they are not designed to predict the MS^2^ spectrum for arbitrary peptides of interest. For better predictive capability, other methods simplify the model by focusing on the prediction of *y*-ion intensities only [8–10]. Although they achieve some success, the applicability of these methods is somewhat restricted.

PeptideART, a data-driven approach based on feed-forward neural networks, is trained with more than 40,000 peptide spectrum matches (PSMs) [11]. In benchmark tests on five different data sets for MS^2^ spectrum prediction, PeptideART compares favorably to MassAnalyzer. MS^2^PIP [12], a later random forest approach, incorporates different predictive models for different peptide lengths (8 to 28 amino acids) and different charge states (charge 2+ and 3+). These models are trained with more than 73,000 PSMs; the overall performance is reported to be better than PeptideART. A web server version of MS^2^PIP has been constructed with a new computational model and a much larger training data set of more than 170,000 PSMs [13]. More recently, a deep neural network-based method called pDeep has been developed [14]. The method is based on a bidirectional long short-term memory (BiLSTM) model and is trained with a data set of around 4,000,000 MS^2^ spectra. Notably, for the same peptide sequence, it predicts MS^2^ spectra of three different fragmentation approaches: HCD (higher-energy collisional dissociation), ETD (electron-transfer dissociation), and EThcD (electron-transfer/higher-energy collision dissociation). According to the reported benchmark experiment, pDeep yields considerable improvements over MassAnalyzer and MS-Simulator.

In this study, we propose MS^2^CNN, a deep convolutional neural network (DCNN) method for MS^2^ spectrum prediction given experimental spectra large enough to effectively train a sophisticated deep learning model. We adopt the network structure of LeNet-5 [15], a DCNN consisting of three major components: a convolutional layer, a pooling layer, and a fully connected layer. A single DCNN is constructed to predict peptides of a specific length and charge. The entire training set was composed of high-quality human HCD MS^2^ spectra from an Orbitrap LC-MS/MS experiment downloaded from the National Institute of Standards and Technology (NIST) consisting of 320,824 unique peptide sequences and 1,127,971 spectra. Five-fold cross validation was performed and the method was then benchmarked on a publicly available independent test dataset of human HeLa cell lysate from LC-MS/MS experiments with MS^2^PIP and pDeep. MS^2^CNN achieved a cosine similarity (COS) in the range of 0.57–0.79 and 0.59–0.74 for peptides of charge 2+ and charge 3+, respectively. These results suggest that MS^2^CNN significantly outperforms MS^2^PIP, especially for shorter peptide sequences for which abundant training data is available. It is also shown that MS^2^CNN has an overall comparable performance to pDeep; however the former predicts MS^2^ spectra for charge 3+ peptides, which are usually considered more complicated than the spectra for 2+ peptides, at a higher accuracy.

## Results

### Five-fold cross validation for determining convolutional layer

Because there are significantly more charge 2+ than charge 3+ peptide sequences, the best layer number of MS^2^CNN is determined by charge 2+, after which the value is directly applied to charge 3+. Given the one-fold run of the five-fold validation result, we chose the 4-layer model as the default structure of MS^2^CNN because it yielded the best performance and is the most efficient of all the models (Additional file 1: Table S4). Although the 5-layer model is comparable to the 4-layer model for some peptide lengths, we did not consider it as its performance fluctuates considerably for peptides of different lengths and it also requires longer training times.

**Fig. 1:**
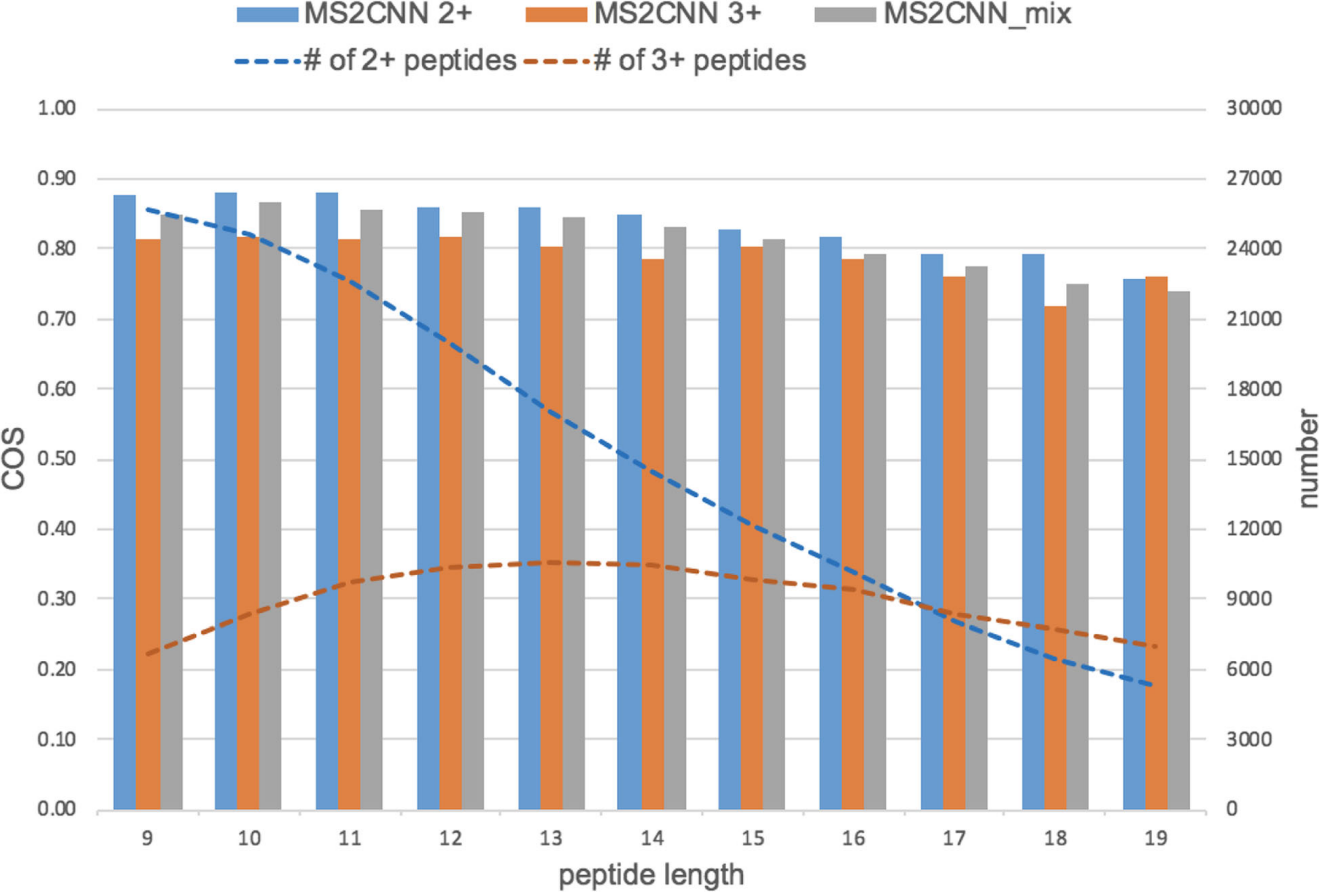
Bar chart of MS^2^CNN COS on charge 2+ (blue), 3+ (orange), and mix (gray) models. Blue and orange dashed lines indicate the peptide number of charge 2+ and 3+ data sets, respectively

Figure 1 shows the five-fold cross validation performance evaluated with COS for different peptide lengths and charge states (other detailed metrics are given in Additional file 1: Table S5). The figure shows that the predictive capability decreases as the peptide length gets larger, possibly due to less training data for longer peptides. We further investigated whether there is a benefit to merging charge 2+ and 3+ training data to build up a single model as MS^2^CNN_mix instead of having the two MS^2^CNN 2+ and MS^2^CNN 3+ models for charge 2+ and charge 3+ peptides, respectively. We followed the previous training procedure with an additional input feature-engineered procedure based on the merged data set of charge 2+ or 3+ peptides. The performance in general falls between the performance of charge 2+ and charge 3+ (Fig. 1, gray bar). This shows that although a larger data set boosts performance (improves MS^2^CNN 3+ performance), different charge states also contain specific patterns in terms of spectrum prediction (impairing MS^2^CNN 2+ performance).

### Upper bound analysis

Peptide fragmentation is a random process; for example, even the same peptide in the same experiment can sometimes result in different peak intensities in spectra. When combining different ionization sources, ion detection, experimental steps, and even different species, the spectrum of the same peptide can be significantly different. Therefore, we compare the similarity between the training spectra and independent spectra for the same peptide sequence (Table 1). Ideally, the similarity in terms of COS or PCC should be 1 if the experimental conditions and the random processes for generating the two spectra are perfectly identical. In reality, the similarity can be seen as the Bayes rate, the theoretical prediction upper bound on prediction accuracy due to unexplainable variance. To conclude, the average upper bound COS for different peptide lengths ranges from 0.600 to 0.800 and decreases as peptide length increases. The average upper bound of PCC for different peptide lengths is even lower, ranging from 0.550 to 0.760. Peptide length seems to have a smaller effect on PCC than on COS, especially for peptides of charge 3 + .

**Table 1:** Average cosine similarity (COS) and Pearson correlation coefficient (PCC) of spectra from the same peptide in training and independent test sets with charge 2+ and charge 3+

Length	Charge 2+		Charge 3+	
	COS	PCC	COS	PCC
9	0.800	0.757	0.617	0.553
10	0.770	0.724	0.781	0.734
11	0.760	0.713	0.771	0.721
12	0.735	0.688	0.735	0.682
13	0.704	0.655	0.732	0.681
14	0.703	0.658	0.703	0.650
15	0.687	0.643	0.672	0.617
16	0.694	0.652	0.691	0.641
17	0.645	0.601	0.690	0.641
18	0.646	0.606	0.660	0.612
19	0.636	0.595	0.668	0.622

### Independent test set evaluation

**Fig. 2:**
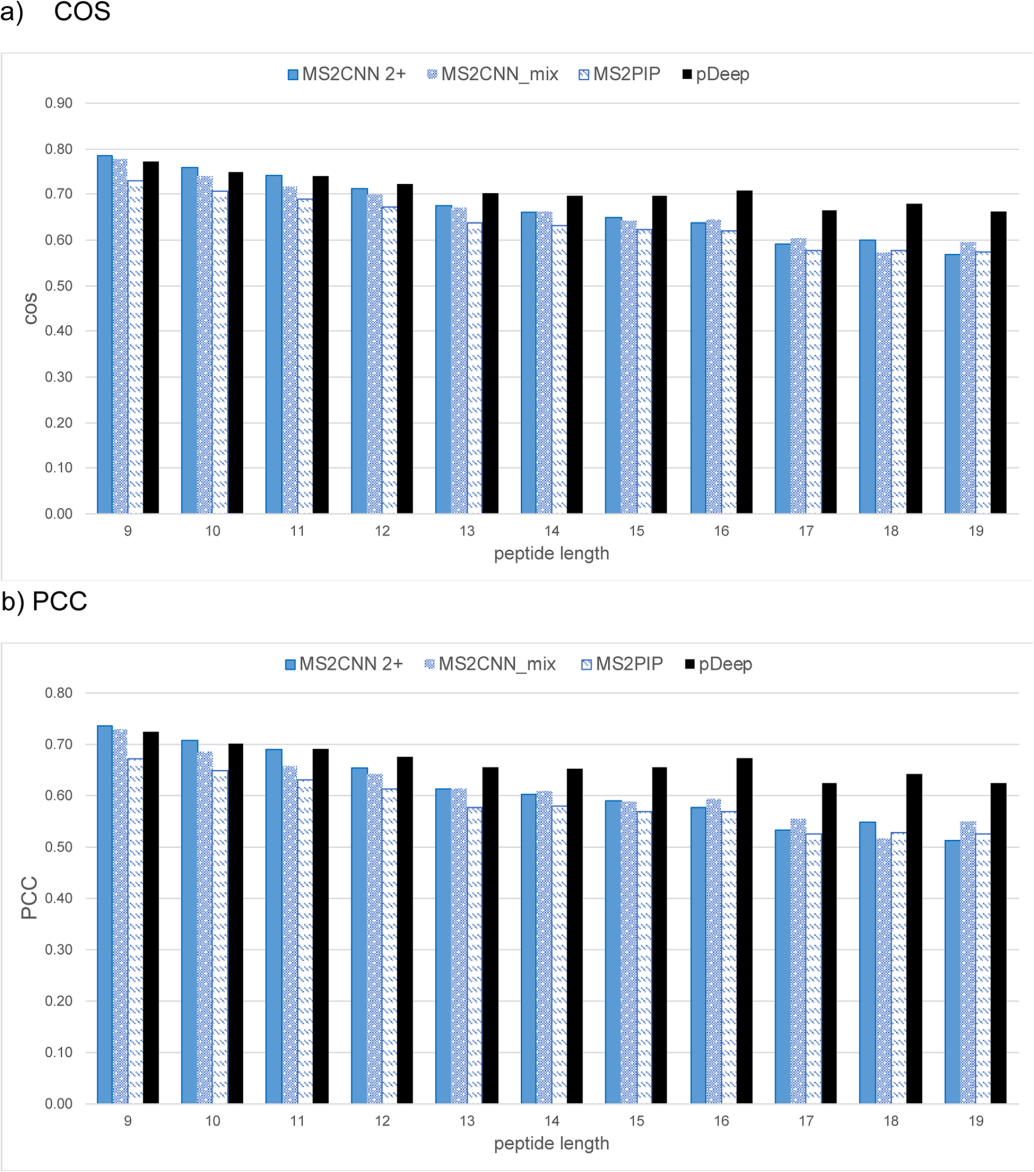
**a** COS (cosine similarity) and **b** PCC (Pearson’s correlation coefficient) of MS^2^CNN 2+ (blue bar), MS^2^CNN_mix (blue bar with white dots), MS^2^PIP (white bar with blue dashes), and pDeep (black bar) on the charge 2+ peptides from the independent test set

**Fig. 3:**
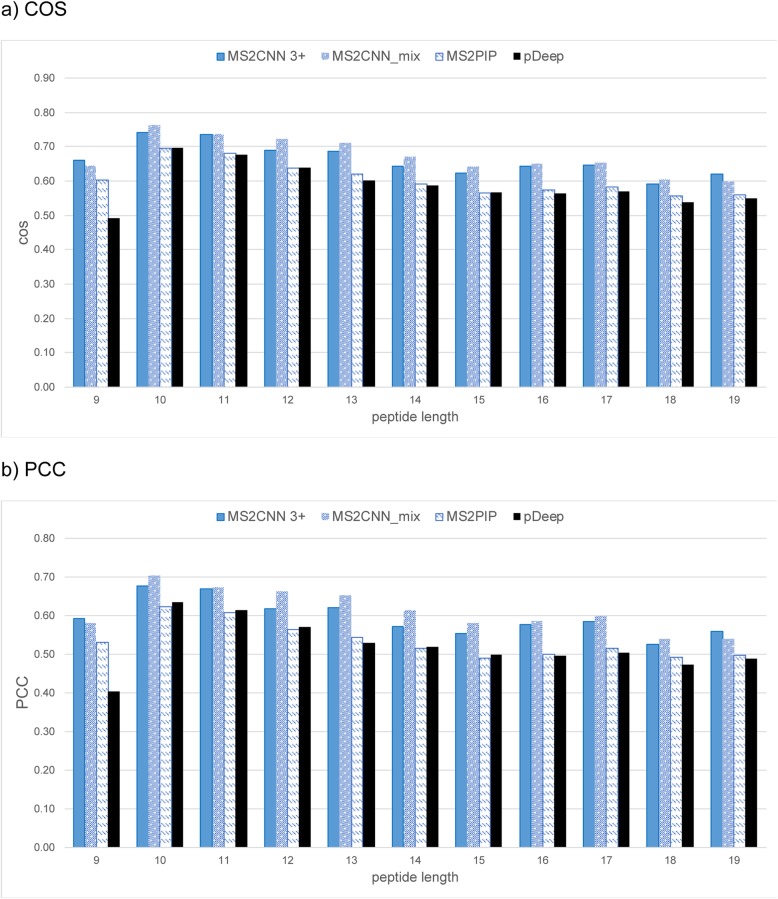
**a** COS and **b** PCC of MS^2^CNN 3+ (blue bar), MS^2^CNN_mix (blue bar with white dots), MS^2^PIP (white bar with blue dashes), and pDeep (black bar) on the charge 3+ peptides from the independent test set

We compared the proposed MS^2^CNN and MS^2^CNN_mix models with MS^2^PIP and pDeep based on the independent test set in terms of COS and PCC (Figs. 2 and 3, detailed values in Additional file 1: Table S6). In general, MS^2^CNN and MS^2^CNN_mix outperform MS^2^PIP for charge 2+ (Fig. 2) and charge 3+ (Fig. 3) peptides in both metrics significantly with a *p*-value < 0.01 by a Wilcoxon signed-rank test (Additional file 2: R Script). For charge 2+ peptides, MS^2^CNN outperforms pDeep marginally for peptide lengths no greater than 11, whereas for peptide lengths from 12 to 19, pDeep considerably outperforms the other methods for both COS and PCC (Fig. 2). In contrast, for charge 3+ peptides, MS^2^CNN and MS^2^CNN_mix yield higher COS and PCC than pDeep for all peptide lengths significantly with a *p*-value < 0.01 by the Wilcoxon signed-rank test (Fig. 3). This suggests that pDeep might be more sensitive to the size of training data, as the number of spectra for charge 3+ peptides is significantly smaller than that of the charge 2+ peptides. Note that pDeep was trained with HCD mouse spectra. Although they show a high MS/MS spectra similarity (a median PCC of 0.94) across different species, a minority of peptides which share low similarity across species can nevertheless deteriorate prediction performance.

Note that the performance of charge 3+ peptides at lengths of 17, 18, and 19 are better than that of charge 2+ peptides for both COS and PCC. This may be due to the richer training data set and higher theoretical prediction upper bound in those ranges. The advantage of MS^2^CNN_mix can be seen in the prediction results of charge 3+ (Fig. 3), for which the size of the training data set greatly increases. This benefit becomes insignificant for charge 2+ peptides, as the original training data set is much larger: the improvement is not affected by theoretical prediction upper bound. Taking charge 3+ peptide lengths of 11 and 12 as an example (Fig. 3 b), there is more improvement in length 12 (MS^2^CNN_mix vs MS^2^PIP) but a higher upper bound in length 11 than length 12 (0.721 vs 0.682, Table 2 charge 3 + .PCC).

**Table 2:** Features used to encode a peptide sequence and its fragment ion sequences

Feature	Description	Package: function name
m/z		Pyteomics v3.4.2: calculate_mass
Original	m/z of the original sequence	
Fragment ion	m/z of the fragment ion sequence	
Isoelectric point	isoelectric point of the sequence	Biopython 1.7: isoelectric_point
Instability index	instability index of the sequence	Biopython 1.7: instability_index
Aromaticity	aromaticity of the sequence	Biopython 1.7: aromaticity
Secondary structure fraction	α-helix, β-strand and coil fraction of the sequence	Biopython 1.7: secondary_structure_fraction
Helicity	helicity of the sequence	In-house program
Hydrophobicity	hydrophobicity of the sequence	in-house program
Basicity	basicity of the sequence	in-house program

## Discussion and conclusion

Peptide identification is an important issue in mass spectrometry-based proteomics. There are two major approaches for peptide identification: database search and spectral library search. Spectral library search boasts a greater sensitivity than database search, but is limited to peptides that have been previously identified. Overcoming this limitation calls for an accurate MS^2^ spectrum prediction tool that is capable of reproducing the chemical fragmentation pattern of a peptide sequence. Over the years, a large number of high quality MS^2^ spectra have been generated and made publicly available by experimentalists, making for an excellent opportunity for researchers to effectively train modern machine learning models such as deep convolutional neural networks for MS^2^ spectra prediction.

We devise DCNN, a deep learning model for the prediction of peak intensities of MS^2^ spectra. In addition to DCNN, we incorporate different Python libraries for feature engineering to facilitate the training process. According to our independent test set of HCD spectra of human samples from Orbitrap LC-MS experiments, MS^2^CNN shows superior prediction performance compared to MS^2^PIP for charge 2+ and 3+ peptides in terms of COS. It also outperforms pDeep, another deep learning approach, for charge 3+ peptides. In the future, we plan to improve the predictive power of our model by either including more data for longer peptide sequences or employing another popular approach in deep learning such as transfer learning, in which a pretrained model is reused for another task, for example, we use a model trained on short peptides for a long peptide task. In the light of our results, we believe MS^2^CNN can be of great use in expanding the coverage of a spectral library and improving the identification accuracy of spectral library search in the analysis of proteomics samples.

## Methods

### Feature engineering

To apply a deep learning method to our dataset, each peptide sequence must be converted into a feature vector with a label. Table 2 lists the features we use to characterize a peptide sequence. These features include peptide composition (similar to amino acid composition), mass-to-charge ratio (m/z), and peptide physical-chemical properties such as isoelectric point, instability index, aromaticity, secondary structure fraction, helicity, hydrophobicity, and basicity. The m/z and physical-chemical features of not only the peptide sequence but all the possible *b* and *y* fragment ions are also included in the feature vector. Take for example the peptide sequence AAAAAAAAGAFAGR (length = 14): its m/z is 577.80, the amino acid composition is {A: 10, C: 0, D: 0, E: 0, F: 1, G: 2, H: 0, I: 0, K: 0, L: 0, M: 0, N: 0, P: 0, Q: 0, R: 1, S: 0, T: 0, V: 0, W: 0, Y: 0}, and the physical-chemical properties {isoelectric point, instability index, aromaticity, helicity, hydrophobicity, basicity, secondary structure fraction} are {9.80, 3.22, 0.07, − 0.21, 1.21, 208.46, (0.071, 0.14, 0.71)}. In addition, the m/z and physical-chemical properties of all the 26 (=2*(14–1)) fragment ions are included in the feature vector. The total number of features for a peptide sequence is 290 (=1 + 20 + 9 + 26*1 + 26*9). We used *Pyteomics* v3.4.2 [16] to compute the mass-to-charge ratio and *Biopython* v1.7 [17] to calculate the amino acid composition, instability index, isoelectric point, and secondary structure fraction.

### MS^2^CNN model

**Fig. 4:**
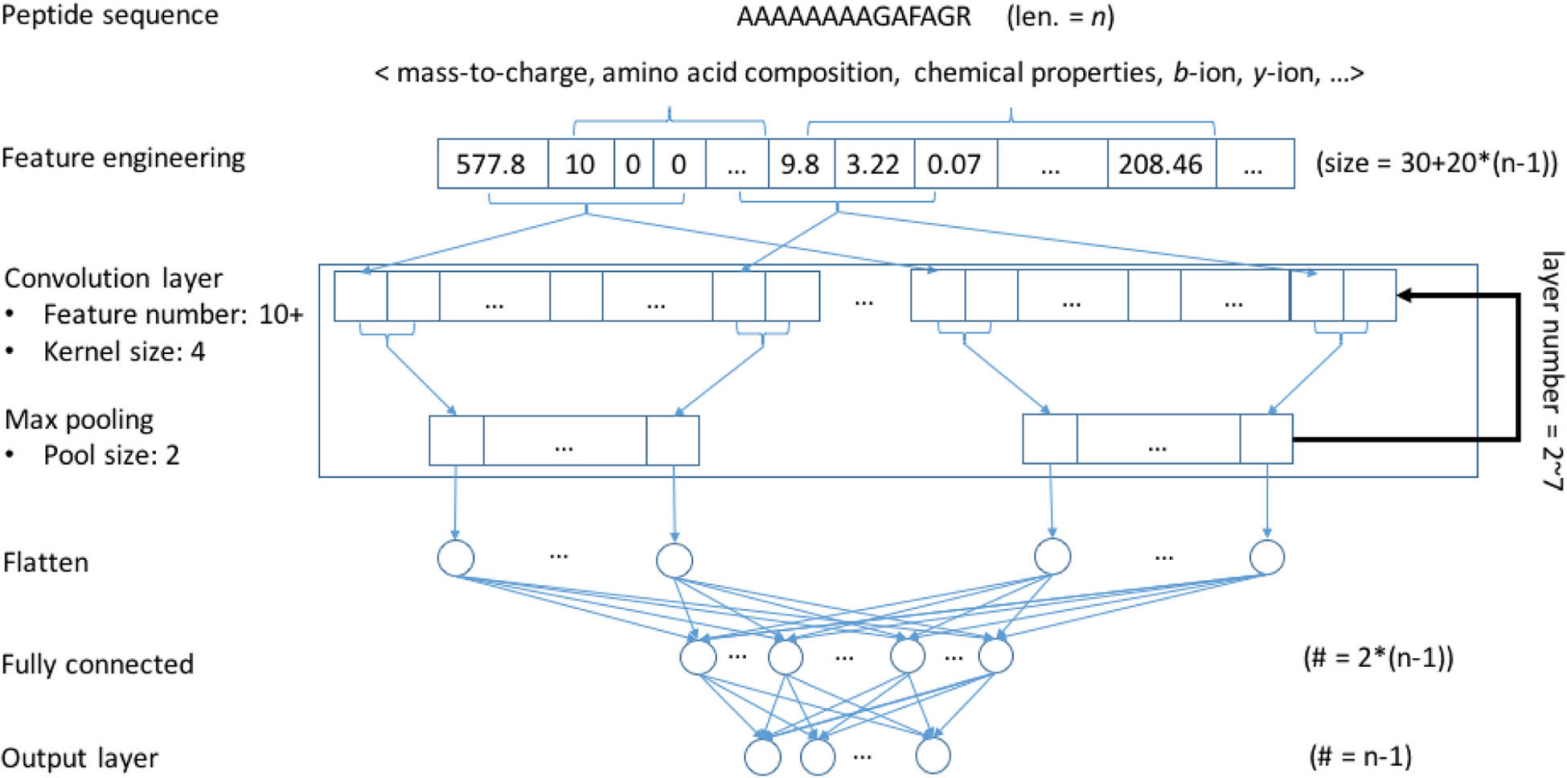
MS^2^CNN model architecture

We propose MS^2^CNN, a DCNN model that uses the aforementioned features (Fig. 4). The MS^2^CNN model takes a peptide feature vector as input and computes an ensemble of nonlinear function nodes in which each layer consists of a number of nodes. The predicted peak intensity corresponds to an output node of the MS^2^CNN model.

In the proposed model, a convolution layer is activated by the *relu* activation function. A max-pooling layer is added after a convolution layer: together they constitute one convolution-pooling layer. The number of convolution-pooling layers is repeated *n* times in MS^2^CNN, where *n* ranges from 2 to 7. The best number was determined by a cross validation experiment. We unify the node number of the convolutional layers as 10; the node number for the last convolutional layer depends on the layer depth. Additional file 1: Table S1 lists the detailed configurations for convolutional layers from layers 2 to 7. The repeated convolution-pooling layers are followed by another layer to flatten the output. Then we add a fully connected layer with twice as many nodes as the number of output nodes. We implemented the MS^2^CNN architecture and executed the whole training process using the *Keras* Python package version 2.0.4 [18]. Figure 4 illustrates the MS^2^CNN model structure.

### Datasets

#### Training data set

We downloaded the training set – a human HCD library based on an Orbitrap mass analyzer and LC-MS (Liquid chromatography–mass spectrometry) – from the NIST website. This set is based on CPTAC and ProteomeXchange, two public repositories containing 1,127,971 spectra from 320,824 unique peptide sequences in *.msp* format. The dataset consists of peptides with charge states ranging from 1+ to 9+, among which only charge states of 2+ and 3+ were selected as there was not enough data for the other charges to effectively train a machine learning model. This strategy is consistent with previous studies.

#### De-duplicated spectrum

It is common for different spectra to belong to the same peptide sequence, and for charge states to have different peak intensities for their fragment ions. We performed a two-step process to generate a de-duplicated spectrum from a set of spectra for a given peptide. First, each peak in a spectrum was normalized by the maximum peak intensity of the spectrum. Then, the intensity of each *b*- and *y*-ion was determined by the median intensity of the ion across different spectra. This yielded a consensus spectrum which filters out noise that could degrade DCNN training. Additional file 1: Table S2 summarizes the number of spectra after deduplication. For effective training of a complex DCNN model, the number of peptides should exceed 5000 after deduplication. Based on this criterion, we focused on peptides of lengths 9 to 19 and eliminated the rest. This resulted in 166,371 charge 2+ peptides (70.4% of the 2+ peptides from NIST) and 98,364 charge 3+ peptides (69.6% of the 3+ peptides from NIST).

#### Independent test set

We used the data-dependent acquisition data of Orbitrap LC-MS experiments from [19] as an independent test set. This included 22,890 and 5998 spectra for charge 2+ and 3+ peptides, respectively. The proportion of common peptides in our training set and independent test set exceeded 90%. Although these peptides were viewed as easier prediction targets, the performance is still bounded by the theoretical upper bound; for example, the upper bound of COS for charge 2+ and charge 3+ peptides ranges from 0.636 to 0.800 and from 0.617 to 0.781, respectively (detailed numbers shown in Table 1). The numbers of commonly observed peptides for different lengths are summarized in Additional file 1: Table S3.

### Evaluation

#### *K*-fold cross validation

To select the best parameters (i.e., layer numbers) for the MS^2^CNN model and to prevent overfitting, we applied five-fold cross validation with a three-way data split, namely, the entire data set was partitioned into training, validation (10% of training data), and test sets. Training epochs continued as long as the accuracy of the validation set improved over the previous epoch by 0.001; otherwise, training was terminated. The final model was selected based on validation performance, and was used to predict the test set for performance evaluation. Since our model was selected based on validation set performance, there was no *data leakage* problem, in which information in the test data is involved in model selection. This problem can result in over-estimation of the performance and unfair comparison with other methods.

#### Metrics

Two metrics are used: *Cosine similarity* (COS) and *Pearson correlation coefficient* (PCC). COS is one of the most widely used spectrum similarity measures for mass spectrometry. It measures the similarity between two non-zero vectors by calculating the angle between them (Eq. 1, calculated by the Python *scikit-learn* package [20]). COS ranges from − 1 to + 1 (angle from 180° to 0°).

1 $$ \mathit{\cos}\left(X,Y\right)=\frac{X{Y}^T}{\left|\left|X\right|\right|\left|\left|Y\right|\right|}\cdots $$

The PCC measures the linear correlation between two variables *X* and *Y* (Eq. 2, calculated by the Python *Scipy* package [21]). It ranges from 1 to − 1, where 1 denotes a completely positive correlation, − 1 a completely negative correlation, and 0 a random correlation or two variables that have no association.

2 $$ {\rho}_{XY}=\frac{\mathit{\operatorname{cov}}\left(X,Y\right)}{\sigma_X\ {\sigma}_Y}\cdots $$

#### Evaluation methods

##### MS^2^PIP

Recently, MS^2^PIP released a new prediction model using XGBoost [22]; the previous random-forest model [13] was not available. Thus, we used the latest MS^2^PIP model for benchmark comparison. The local standalone version (Python code downloaded from [23]) was used instead of the online server as the latter is subject to a maximum number of 5000 peptides per query.

We used the default settings of MS^2^PIP according to the Github config file, other than changing *frag_method* from HCD to HCDch2. In addition, the MGF function was enabled to generate intensities without log_2_ transformation. To ensure a fair comparison, we processed the test data using the same peak normalization procedure used to process our training data.

##### pDeep

First, we converted a peptide to a 2D array using the pDeep API. Then, we loaded the pDeep model (.h5 format), which we used to predict the intensities of the peptide [14]. Although the pDeep documentation states “If the precursor charge state is <= 2, 2+ ions should be ignored”, to ensure a fair and complete charge 2+ peptide comparison, we set the intensity of the testing 2+ peak to zero as if it were missing in pDeep prediction. pDeep provided three trained models – BiLSTM, ProteomeTools-ETD, and ProteomeTools-EThcD – of which the BiLSTM model was used for comparison as it performed the best in both COS and PCC metrics (Additional file 1: Table S6).

## Supplementary information

**Additional file 1:** Excel file: Supplementary tables S1–S6 for additional results.

**Additional file 2:** R Script: Wilcoxon signed-rank test for independent test set evaluation.

## Data Availability

Our source code for the whole experiments, including preprocessing, feature engineering, and MS^2^CNN, is publicly available at https://github.com/changlabtw/MS2CNN. The materials generated and analyzed during the current study are available at ○ Training data https://figshare.com/s/52fcaade571a7d60241c. ○ Independent test data https://figshare.com/s/91e99d2c9f7ce03f1210.

## Abbreviations

COS: Cosine similarity
DCNN: Deep convolutional neural network
KNN: *K*-nearest neighbor
m/z: mass-to-charge
MS: Mass spectrometry
MS^2^: Tandem mass spectrometry
PCC: Pearson correlation coefficient
